# STRENGTHENING A COMMUNITY HEALTHY AGING EDUCATION PROGRAM THROUGH CBO PARTNERSHIPS AND RCQI

**DOI:** 10.1093/geroni/igz038.2466

**Published:** 2019-11-08

**Authors:** Jeffrey P Graupner, Jason Molony, Leah Kelemen, Kedong Ding, Katherine Thompson

**Affiliations:** 1 University of Chicago, Chicago, Illinois, United States; 2 University of Chicago School of Social Service Administration, Chicago, Illinois, United States

## Abstract

The SHARE Network (Supporting Healthy Aging Resources & Education) is a Geriatrics Workforce Enhancement Program serving older adults in a large urban, underserved community. For 4 years, one objective of the SHARE Network has been to increase health literacy while fostering positive change in older adults through “Healthy Aging” presentations. Led by geriatrics subject experts, these presentations cover a wide range of important health topics for older adults at a variety of community-based organization (CBO) partner locations. Rapid Cycle Quality Improvement (RCQI) efforts to enhance this programming include surveying audience members after a presentation, then sharing results with CBO partners at monthly meetings to review popular topics, participant demographics, quality of presentations, suggestions for improvement, etc. These recurring RCQI meetings have proven critical to maintaining a robust healthy aging curriculum and network engagement across a variety of organizations and interests. Analysis of participant survey data demonstrates the strength of QI efforts and includes a qualitative analysis of open-ended responses. To date, 153 presentations have been held with 2,704 attendees and 1,470 respondents, 86.3% of whom planned to make a health change after attending the presentation. Qualitative analysis of these behavior change plans reveals that 82.6% are proactive behaviors versus restrictive behaviors, and that diet, exercise, and lifestyle changes are most common. This program, and its RCQI approach, serves as a successful model for aligning the educational objectives and priorities of network partners, while ensuring relevancy and cultural competency in promoting positive healthy behaviors in the older adult community.

